# Supplementary material for: Detection of maternal transmission of resistant Gram-negative bacteria in a Cambodian hospital setting
Source: Front Microbiol. 2023 Apr 14;14:1158056. doi: 10.3389/fmicb.2023.1158056 (PMC10140293; doi:10.3389/fmicb.2023.1158056)
Supplement: Supplementary file 1 [file Data_Sheet_1.PDF]

Supplementary Figures

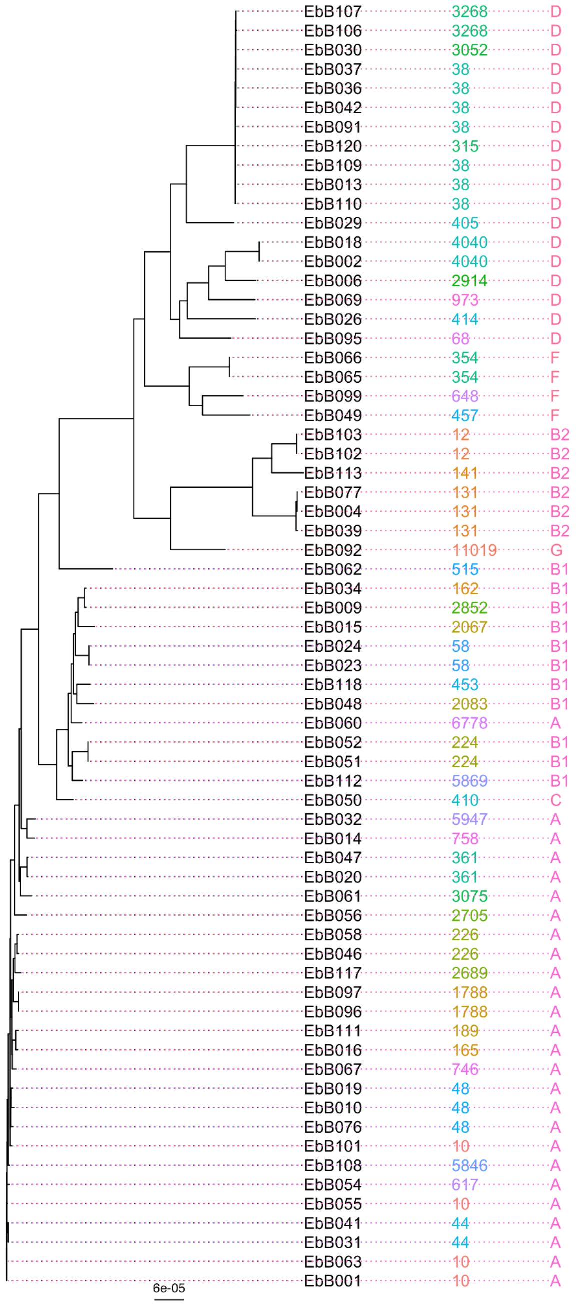

Supplementary Figure 1. A phylogeny of the *E. coli* isolates sequenced in this study including their ST and phylogroup. Variant calls from Snippy were filtered to remove repeat regions and recombinant regions using Gubbins, and the core SNPs were used to generate a tree using IQ-TREE.

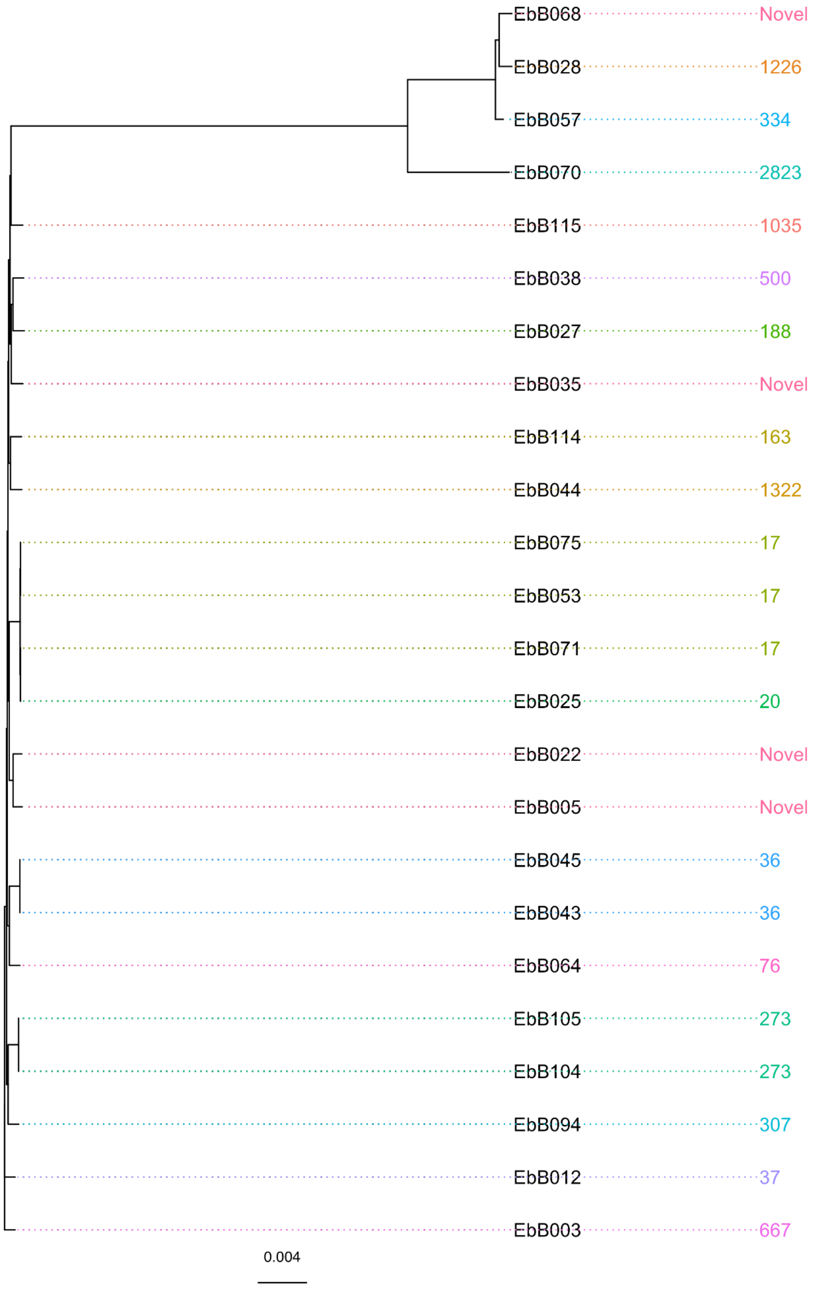

Supplementary Figure 2. A phylogeny of the *K. pneumoniae* isolates sequenced in this study including their ST. Variant calls from Snippy were filtered to remove repeat regions and recombinant regions using Gubbins, and the core SNPs were used to generate a tree using IQ-TREE.

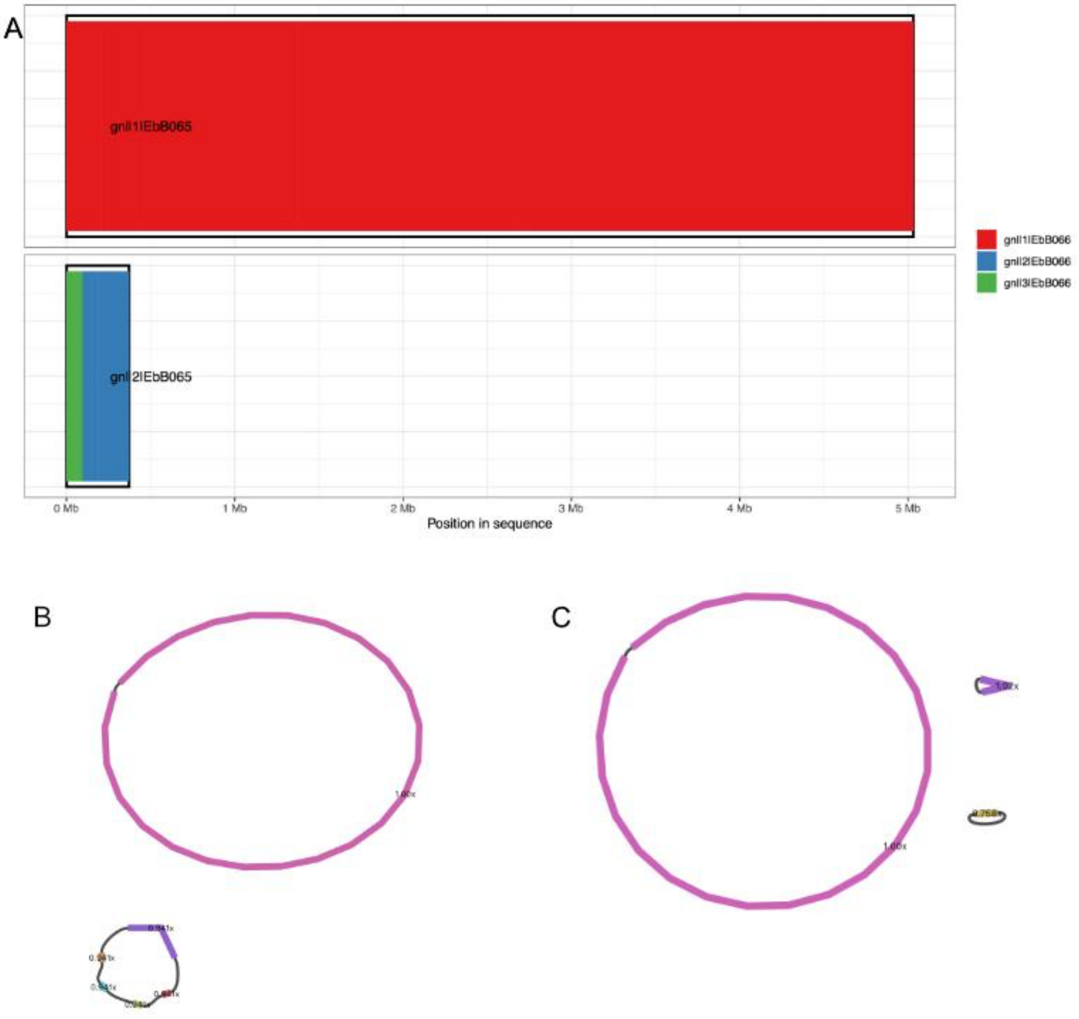

Supplementary Figure 3. A) Alignment between the three contigs assembled in strain EbB066, represented by different coloured blocks as shown in the legend, and the two contigs assembled in strain EbB065. The two non-chromosomal contigs in strain EbB066 are present as one contig in strain EbB065. Bandage visualization of the assembly graph for B) EbB065 and C) EbB066.

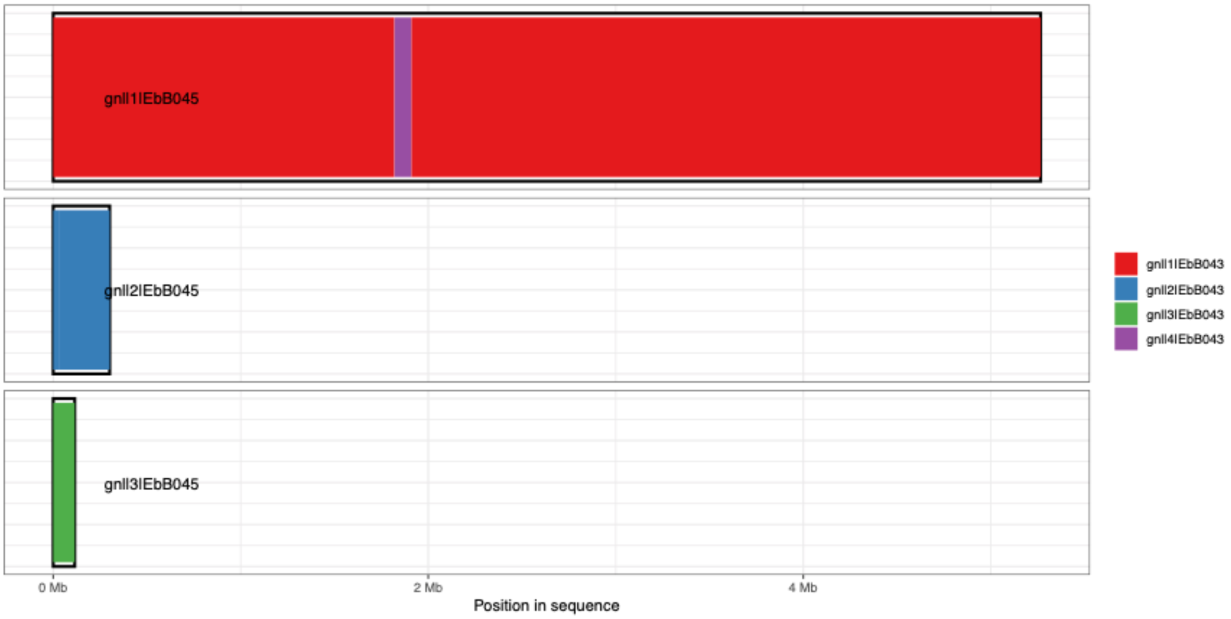

Supplementary Figure 4. A) Alignment between the chromosome and three contigs assembled in strain EbB043, represented by different coloured blocks as shown in the legend, and the chromosome and two contigs assembled in strain EbB045. A non-circular contig assembled in EbB043 is inserted into the chromosome of EbB045.

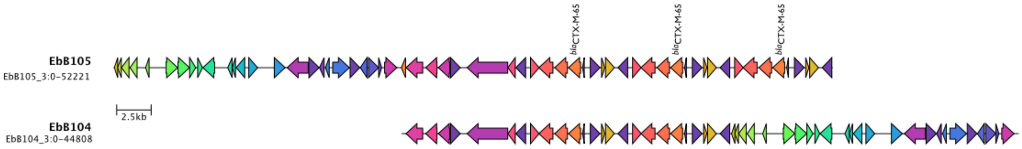

Supplementary Figure 5. Alignment of plasmid region of EbB104 and EbB105 strains showing the tandem duplication of the *bla*$_{\text{CTX-M-65}}$ gene (labelled) seen in two copies in EbB104 and three copies in EbB105.
